# Supplementary material for: Short-Form Thymic Stromal Lymphopoietin (sfTSLP) Is the Predominant Isoform Expressed by Gynaecologic Cancers and Promotes Tumour Growth
Source: Cancers (Basel). 2021 Feb 26;13(5):980. doi: 10.3390/cancers13050980 (PMC7956741; doi:10.3390/cancers13050980)
Supplement: Supplementary file 1 [file cancers-13-00980-s001.pdf]

Figure S1

Homo sapiens thymic stromal lymphopoietin (TSLP), transcript variant 1, mRNA
NCBI Reference Sequence: NM_033035.4

Keys:

[Coding sequence];
Primer binding sites;
PCR target region (TSLPv1) flanked by primers designed by us;
PCR target region (CDS of TSLPv1) flanked by primers designed by us;
PCR target region (lfTSLP) flanked by primers designed by Fornasa et al.(29) and Biancheri et al.(30);
{Binding site of Basescope probe};
Sequence that overlapped with Homo sapiens thymic stromal lymphopoietin (TSLP), transcript variant 2, mRNA (NM_138551.4)

1 gcagccagaa agctctggag catcagggag actccaactt aaggcaacag catgggtgaa
61 taaggg{cttc ctgtggactg gcaatgagag gcaaaacctg gtgcttgagc actggcccct
121 aaggcaggcc ttacagatct cttacactcg} tggtgggaag agtttagtgt gaaactgggg
181 tggaattggg tgtccacgt[a tgttcccttt tgccttacta tatgttctgt cagtttcttt
241 caggaaaatc ttcatcttac aacttgtagg gctggtgtta acttacgact tcactaactg
301 tgactttgag aagattaaag cagcctatct cagtactatt tctaaagacc tgattacata
361 tatgagtggg accaaaagta ccgagttcaa caacaccgtc tcttgtagca atcg***gccaca***
421 ttgccttact gaaatccaga gcctaacctt caatcccacc gccggctgcg cgtcgctcgc
481 caaagaaatg ttcgccatga aaactaaggc tgccttagct atctggtgcc caggctattc
541 ggaaactcag ataaatgcta ctcaggcaat gaagaagagg agaaaaagga aagtcacaac
601 caataaatgt ctggaacaag tgtcacaatt acaaggattg tggcgtcgct tcaatcgacc
661 tttactgaaa caacagtaa]a ccatctttat tatggtcata tttcacagca ccaaaataaa
721 tcatctttat taagtagatg aaacattaac tctaactgtg acaaagaaga ccacaaatag
781 ttatctttta attacagaag agtttcttaa cttacttttg taagttttta ttgtgtaagt
841 ttataatgca ggggaagtac tactcctcaa atgttgaggg aagcttccat aacattgatg
901 actggcttca tggcagtaat tctcggctgt agttgcataa gcattgctca agaggaaaat
961 ccaaaagtgc agcaggagaa ctcttttccc tgaaaaagga aaaatattga actcaatgat
1021 agcacctaaa cttacattta aaagacagac attccttcta catgtaatga cacttcttgt
1081 gttaaactaa aaatttacaa gagaagaaag tgaaagcaaa tggggtttca caaatagttg
1141 taaatatagt gaagcaattt gaaataattt tcaagcaaag tattgtgaaa gtattctaag
1201 ccaagtttta aatattatct aacagacaag agtggtatat acaagtagat cctgagaagt
1261 acctttgtta cagctactat aaatatacat ataaattata gaatctactt taatttattt
1321 tgtgaacact tttgaaaatg tacatgttcc tttgtaattg acactatata tttcttaata
1381 aaataattct caaatttgtt tcttatgaat catctctcaa atctagttag acaatttgca
1441 cacatacttt tctaagggac attatcttcc ttcaggtttt tacctccact catccttaga
1501 gcccactgac tgctcccctt tatacctgtt ggccctgcct ataggagaga atatttggag
1561 ataggcagct tcaggatgca ttgcaatcat ccttttctta aattatgtca ctagtctttt
1621 atttttttccc ctcttgaact ttcctcacac ctggaagaaa caaagtagga aaaagtgaac
1681 aggggatgtc aaatcgattc ttgaattccc gctgcaagct agagccgcag gcaccctctc
1741 actcaatttc cactcagaac cctataaaca ccagtgggaa gggcaaccca ctgcacgtgg
1801 gaatgcactg atttttccta ggagtagaca tgttcctcta attactccct gagggttagt
1861 tggggctaaa ccatgacaga agtggggaag ttcaatgtcc ttaaatccat cttacttgcc
1921 aacaggtaag aggaagctta cattacatgt ccagtccaca tttaaagagc acttactgtg
1981 gaacaagcct tcagccaaac aatggggata gaaaagtagg taagactcag cctttgtcca
2041 gagaagctca gggtatagct gaataggcag tttcttttgt cctgaggaaa atcaggacat
2101 gcctgctttc taaaaatctt cctctgaaga cctgacccaa gctcttaaat gctattgtaa
2161 gagaaatttc tttgtctatt aactccattt tagtagggat tcactgacta gattttactg
2221 aactatgaaa ataaatacac ataattttc acaaaatttt gggcccaatt cccctaaaag
2281 aattgaggat tagggagaaa ggagacaact caaagtcatc ccattaagtg cagtttcttt
2341 gaatcttctg ctttatcttt aaaaatttgt ataatttata tattttattc tatgtgttcc
2401 atagatatct taatgtaaaa ttagtcattt aaattacact gtcaattaaa agtaatgggc
2461 aagagattgc atcatactaa tttagtaaga acgttcccaa atgttgtaac aatgtggatc
2521 atacatctct ggttttttaa atgtattgag gctttcttgg tggactagta tagtatacgg
2581 tcagttatgt caatgtttca tggtcaataa aaaggaagtt gcaaattgt

1 gcagccagaa agctctggag catcagggag actccaactt aaggcaacag catgggtgaa
61 taaggg{cttc ctgtggactg gcaatgagag gcaaaacctg gtgcttgagc actggcccct
121 aaggcaggcc ttacagatct cttacactcg} tggtgggaag agtttagtgt gaaactgggg
181 tggaattggg tgtccacgt[a tgttcccttt tgccttacta tatgttctgt cagtttcttt
241 caggaaaatc ttcatcttac aacttgtagg gctggtgtta acttacgact tcactaactg
301 tgactttgag aagattaaag cagcctatct cagtactatt tctaaagacc tgattacata
361 tatgagtggg accaaaagta ccgagttcaa caacaccgtc tcttgtagca atcg***gccaca***
421 ttgccttact gaaatccaga gcctaacctt caatcccacc gccggctgcg cgtcgctcgc
481 caaagaaatg ttcgccatga aaactaaggc tgccttagct atctggtgcc caggctattc
541 ggaaactcag ataaatgcta ctcaggcaat gaagaagagg agaaaaagga aagtcacaac
601 caataaatgt ctggaacaag tgtcacaatt acaaggattg tggcgtcgct tcaatcgacc
661 tttactgaaa caacagtaa]a ccatctttat tatggtcata tttcacagca ccaaaataaa
721 tcatctttat taagtagatg aaacattaac tctaactgtg acaaagaaga ccacaaatag
781 ttatctttta attacagaag agtttcttaa cttacttttg taagttttta ttgtgtaagt
841 ttataatgca ggggaagtac tactcctcaa atgttgaggg aagcttccat aacattgatg
901 actggcttca tggcagtaat tctcggctgt agttgcataa gcattgctca agaggaaaat
961 ccaaaagtgc agcaggagaa ctcttttccc tgaaaaagga aaaatattga actcaatgat
1021 agcacctaaa cttacattta aaagacagac attccttcta catgtaatga cacttcttgt
1081 gttaaactaa aaatttacaa gagaagaaag tgaaagcaaa tggggtttca caaatagttg
1141 taaatatagt gaagcaattt gaaataattt tcaagcaaag tattgtgaaa gtattctaag
1201 ccaagtttta aatattatct aacagacaag agtggtatat acaagtagat cctgagaagt
1261 acctttgtta cagctactat aaatatacat ataaattata gaatctactt taatttattt
1321 tgtgaacact tttgaaaatg tacatgttcc tttgtaattg acactatata tttcttaata
1381 aaataattct caaatttgtt tcttatgaat catctctcaa atctagttag acaatttgca
1441 cacatacttt tctaagggac attatcttcc ttcaggtttt tacctccact catccttaga
1501 gcccactgac tgctcccctt tatacctgtt ggccctgcct ataggagaga atatttggag
1561 ataggcagct tcaggatgca ttgcaatcat ccttttctta aattatgtca ctagtctttt
1621 atttttttccc ctcttgaact ttcctcacac ctggaagaaa caaagtagga aaaagtgaac
1681 aggggatgtc aaatcgattc ttgaattccc gctgcaagct agagccgcag gcaccctctc
1741 actcaatttc cactcagaac cctataaaca ccagtgggaa gggcaaccca ctgcacgtgg
1801 gaatgcactg atttttccta ggagtagaca tgttcctcta attactccct gagggttagt
1861 tggggctaaa ccatgacaga agtggggaag ttcaatgtcc ttaaatccat cttacttgcc
1921 aacaggtaag aggaagctta cattacatgt ccagtccaca tttaaagagc acttactgtg
1981 gaacaagcct tcagccaaac aatggggata gaaaagtagg taagactcag cctttgtcca
2041 gagaagctca gggtatagct gaataggcag tttcttttgt cctgaggaaa atcaggacat
2101 gcctgctttc taaaaatctt cctctgaaga cctgacccaa gctcttaaat gctattgtaa
2161 gagaaatttc tttgtctatt aactccattt tagtagggat tcactgacta gattttactg
2221 aactatgaaa ataaatacac ataatttttc acaaaatttt gggcccaatt cccctaaaag
2281 aattgaggat tagggagaaa ggagacaact caaagtcatc ccattaagtg cagtttcttt
2341 gaatcttctg ctttatcttt aaaaatttgt ataatttata tattttattc tatgtgttcc
2401 atagatatct taatgtaaaa ttagtcattt aaattacact gtcaattaaa agtaatgggc
2461 aagagattgc atcatactaa tttagtaaga acgttcccaa atgttgtaac aatgtggatc
2521 atacatctct ggttttttaa atgtattgag gctttcttgg tggactagta tagtatacgg
2581 tcagttatgt caatgtttca tggtcaataa aaaggaagtt gcaaattgt

Figure S2

Homo sapiens thymic stromal lymphopoietin (TSLP), transcript variant 2, mRNA
NCBI Reference Sequence: NM_138551.4

Keys:

[Coding sequence];
Primer binding sites;
PCR target region (TSLPv2) flanked by primers designed by us;
PCR target region (CDS of TSLPv2) flanked by primers designed by us;
PCR target region (sfTSLP) flanked by primers designed by Fornasa et al.(29) and Biancheri et al.(30);
{Binding site of Basescope probe};
Sequence that overlapped with Homo sapiens thymic stromal lymphopoietin (TSLP), transcript variant 1, mRNA (NM_033035.4)

1 accctcgcca cgcccctgct cccccgcggt t{ggttcttcc ttgctctact caaccctgac
61 ctcttctctc tgactctcga cttgtgttcc ccgctcctcc ctgaccttcc tcccctcccc
121 tttcactcaa ttctcaccaa ctctttctct ctctggtgtt ttctcc}tttt ctcgtaaact
181 ttgccgccta tgagca***gcca cattgcctta ctgaaatcca gagcctaacc ttcaatccca***
241 ccgccggctg cgcgtcgctc gccaaagaa[***a tgttcgccat gaaaactaag gctgccttag***
301 ctatctggtg cccaggctat tcggaaactc agataaatgc tactcaggca atgaagaaga
361 ggagaaaaag gaaagtcaca accaataaat gtctggaaca agtgtcacaa ttacaaggat
421 tgtggcgtcg cttcaatcga cctttactga aacaacagta a]***accatcttt attatggtca***
481 tatttcacag caccaaaata aatcatcttt attaagtaga tgaaacatta actctaactg
541 tgacaaagaa gaccacaaat agttatcttt taattacaga agagtttctt aacttacttt
601 tgtaagtttt tattgtgtaa gtttataatg caggggaagt actactcctc aaatgttgag
661 ggaagcttcc ataacattga tgactggctt catggcagta attctcggct gtagttgcat
721 aagcattgct caagaggaaa atccaaaagt gcagcaggag aactcttttc cctgaaaaag
781 gaaaaatatt gaactcaatg atagcaccta aacttacatt taaaagacag acattccttc
841 tacatgtaat gacacttctt gtgttaaact aaaaatttac aagagaagaa agtgaaagca
901 aatggggttt cacaaatagt tgtaaatata gtgaagcaat ttgaaataat tttcaagcaa
961 agtattgtga aagtattcta agccaagttt taaatattat ctaacagaca agagtggtat
1021 atacaagtag atcctgagaa gtacctttgt tacagctact ataaatatac atataaatta
1081 tagaatctac tttaatttat tttgtgaaca cttttgaaaa tgtacatgtt cctttgtaat
1141 tgacactata tatttcttaa taaaataatt ctcaaatttg tttcttatga atcatctctc
1201 aaatctagtt agacaatttg cacacatact tttctaaggg acattatctt ccttcaggtt
1261 tttacctcca ctcatcctta gagcccactg actgctcccc tttatacctg ttggccctgc
1321 ctataggaga gaatatttgg agataggcag cttcaggatg cattgcaatc atccttttct
1381 taaattatgt cactagtctt ttattttttc ccctcttgaa ctttcctcac acctggaaga
1441 aacaaagtag gaaaaagtga acaggggatg tcaaatcgat tcttgaattc ccgctgcaag
1501 ctagagccgc aggcaccctc tcactcaatt tccactcaga accctataaa caccagtggg
1561 aagggcaacc cactgcacgt gggaatgcac tgatttttcc taggagtaga catgttcctc
1621 taattactcc ctgagggtta gttggggcta aaccatgaca gaagtgggga agttcaatgt
1681 ccttaaatcc atcttacttg ccaacaggta agaggaagct tacattacat gtccagtcca
1741 catttaaaga gcacttactg tggaacaagc cttcagccaa acaatgggga tagaaaagta
1801 ggtaagactc agcctttgtc cagagaagct cagggtatag ctgaataggc agtttctttt
1861 gtcctgagga aaatcaggac atgcctgctt tctaaaaatc ttcctctgaa gacctgaccc
1921 aagctcttaa atgctattgt aagagaaatt tctttgtcta ttaactccat tttagtaggg
1981 attcactgac tagattttac tgaactatga aaataaatac acataatttt tcacaaaatt
2041 ttgggcccaa ttccccctaaa agaattgagg attagggaga aaggagacaa ctcaaagtca
2101 tcccattaag tgcagtttct ttgaatcttc tgctttatct ttaaaaattt gtataattta
2161 tatattttat tctatgtgtt ccatagatat cttaatgtaa aattagtcat ttaaattaca
2221 ctgtcaatta aaagtaatgg gcaagagatt gcatcatact aatttagtaa gaacgttccc
2281 aaatgttgta acaatgtgga tcatacatct ctggtttttt aaatgtattg aggctttctt
2341 ggtggactag tatagtatac ggtcagttat gtcaatgttt catggtcaat aaaaaggaag
2401 ttgcaaattg t

1 accctcgcca cgcccctgct cccccgcggt t{ggttcttcc ttgctctact caaccctgac
61 ctcttctctc tgactctcga cttgtgttcc ccgctcctcc ctgaccttcc tcccctcccc
121 tttcactcaa ttctcaccaa ctctttctct ctctggtgtt ttctcc}tttt ctcgtaaact
181 ttgccgccta tgagca***gcca cattgcctta ctgaaatcca gagcctaacc ttcaatccca***
241 ccgccggctg cgcgtcgctc gccaaagaa[a tgttcgccat gaaaactaag gctgccttag
301 ctatctggtg cccaggctat tcggaaactc agataaatgc tactcaggca atgaagaaga
361 ggagaaaaag gaaagtcaca accaataaat gtctggaaca agtgtcacaa ttacaaggat
421 tgtggcgtcg cttcaatcga cctttactga aacaacagta a]accatcttt attatggtca
481 tatttcacag caccaaaata aatcatcttt attaagtaga tgaaacatta actctaactg
541 tgacaaagaa gaccacaaat agttatcttt taattacaga agagtttctt aacttacttt
601 tgtaagtttt tattgtgtaa gtttataatg caggggaagt actactcctc aaatgttgag
661 ggaagcttcc ataacattga tgactggctt catggcagta attctcggct gtagttgcat
721 aagcattgct caagaggaaa atccaaaagt gcagcaggag aactcttttc cctgaaaaag
781 gaaaaatatt gaactcaatg atagcaccta aacttacatt taaaagacag acattccttc
841 tacatgtaat gacacttctt gtgttaaact aaaaatttac aagagaagaa agtgaaagca
901 aatggggttt cacaaatagt tgtaaatata gtgaagcaat ttgaaataat tttcaagcaa
961 agtattgtga aagtattcta agccaagttt taaatattat ctaacagaca agagtggtat
1021 atacaagtag atcctgagaa gtacctttgt tacagctact ataaatatac atataaatta
1081 tagaatctac tttaatttat tttgtgaaca cttttgaaaa tgtacatgtt cctttgtaat
1141 tgacactata tatttcttaa taaaataatt ctcaaatttg tttcttatga atcatctctc
1201 aaatctagtt agacaatttg cacacatact tttctaaggg acattatctt ccttcaggtt
1261 tttacctcca ctcatcctta gagcccactg actgctcccc tttatacctg ttggccctgc
1321 ctataggaga gaatatttgg agataggcag cttcaggatg cattgcaatc atccttttct
1381 taaattatgt cactagtctt ttatttttc ccctcttgaa ctttcctcac acctggaaga
1441 aacaaagtag gaaaaagtga acaggggatg tcaaatcgat tcttgaattc ccgctgcaag
1501 ctagagccgc aggcaccctc tcactcaatt tccactcaga accctataaa caccagtggg
1561 aagggcaacc cactgcacgt gggaatgcac tgatttttcc taggagtaga catgttcctc
1621 taattactcc ctgagggtta gttggggcta aaccatgaca gaagtgggga agttcaatgt
1681 ccttaaatcc atcttacttg ccaacaggta agaggaagct tacattacat gtccagtcca
1741 catttaaaga gcacttactg tggaacaagc cttcagccaa acaatgggga tagaaaagta
1801 ggtaagactc agcctttgtc cagagaagct cagggtatag ctgaataggc agtttctttt
1861 gtcctgagga aaatcaggac atgcctgctt tctaaaaatc ttcctctgaa gacctgaccc
1921 aagctcttaa atgctattgt aagagaaatt tctttgtcta ttaactccat tttagtaggg
1981 attcactgac tagattttac tgaactatga aaataaatac acataatttt tcacaaaatt
2041 ttgggcccaa ttcccctaaa agaattgagg attagggaga aaggagacaa ctcaaagtca
2101 tcccattaag tgcagtttct ttgaatcttc tgctttatct ttaaaaattt gtataattta
2161 tatattttat tctatgtgtt ccatagatat cttaatgtaa aattagtcat ttaaattaca
2221 ctgtcaatta aaagtaatgg gcaagagatt gcatcatact aatttagtaa gaacgttccc
2281 aaatgttgta acaatgtgga tcatacatct ctggtttttt aaatgtattg aggctttctt
2341 ggtggactag tatagtatac ggtcagttat gtcaatgttt catggtcaat aaaaaggaag
2401 ttgcaaattg t

a

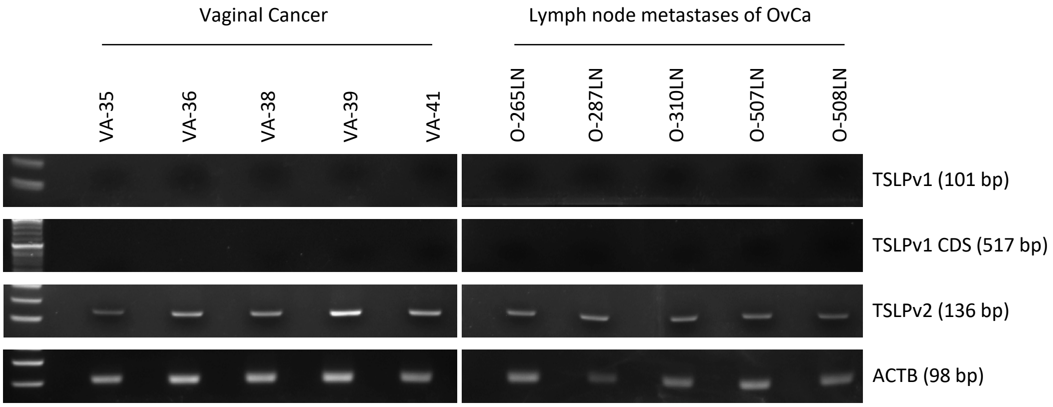

b

Sanger Sequencing (VA-41)

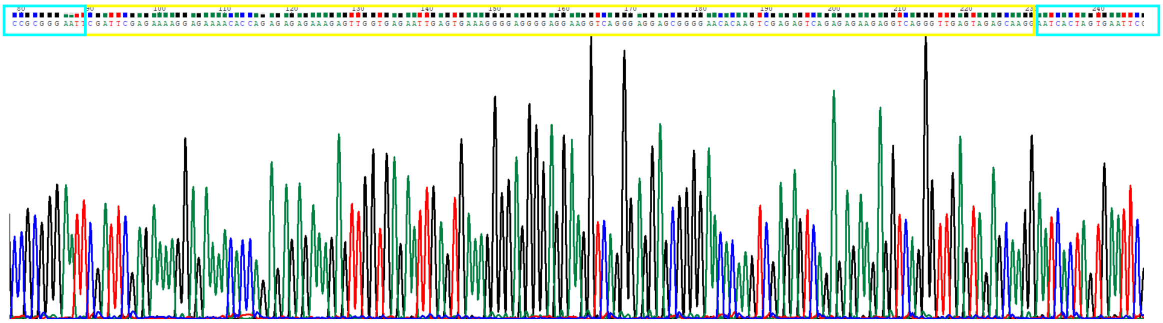

AAACGCCCGCTGCATTGTATACGACTCACTATAGGGCGAATTGGGCCCGACGTCGCATGCTCCCGGCCGCCATGGCGGCCGCGGGAATTCGAT
TCGAGAAAAGGAGAAAACACCAGAGAGAGAAAGAGTTGGTGAGAATTGAGTGAAAGGGGAGGGGAGGAAGGTCAGGGAGGAGCGGGGAA
CACAAGTCGAGAGTCAGAGAGAAGAGGTCAGGGTTGAGTAGAGCAAGGAATCACTAGTGAATTCGCGGCCGCCTGCAGGTCGACCATATGG
GAGAGCTCCCAACGCGTTGGATGCATAGCTTGAGTATTCTATAGTGTCACCTAAATAGCTTGGCGTAATCATGGTCATAGCTGTTTCCTGTGTGA
AATTGTTATCCGCTCACAATTCCACACAACATACGAGCCGGAAGCATAAAGTGTAAAGCCTGGGGTGCCTAATGAGTGAGCTAACTCACATTAA
TTGCGTTGCGCTCACTGCCCGCTTTCCAGTCGGGAAACCTGTCGTGCCAGCTGCATTAATGAATCGGCCAACGCGCGGGGAGAGGCGGTTTGC
GTATTGGGCGCTCTTCCGCTTCCTCGCTCACTGACTCGCTGCGCTCGGTCGTTCGGCTGCGGCGAGCGGTATCAGCTCACTCAAAGGCGGTAAT
ACGGTTATCCACAGAATCAGGGGATAACGCAGGAAAGAACATGTGAGCAAAAGGCCAGCAAAAGGCCAGGAACCGTAAAAAGGCCGCGTTG
CTGGCGTTTTTCCATAGGCTCCGCCCCCCTGACGAGCATCACAAAAATCGACGCTCAAGTCAGAGGTGGCGAAACCCGACAGGACTATAAAG
ATACCAGGCGTTTCCCCCTGGAAGCTCCCTCGTGCGCTCTCCTGTTCCGACCCTGCCGCTTACCGGATACCTGTCCGCCTTTCTCCCTTCGGAAGC
GTGCGCTTTCTCATAGCTCACGCTGTAGTATCTCAGTTTCGGTGTAGGTCGTTCGCTCAAGCTGGGCTGTGTGCACGAACCCCCCGTTCAGGCC
CGACCGGCTGCGCCTTATCAGTAACTATCGTCTGAGTTCGACCGGTAGACCGACTATCGCACTGGCAGCAGTCTCACCTGTGGTAC

Product of RT-PCR of TSLPv2 (reverse)
pGEM-T Easy vector

Fig. S3 **RT-PCR analysis of TSLPv1 and TSLPv2 in vaginal cancer tissues and lymph node metastases of ovarian cancer.** (a) RT-PCR of TSLPv1, CDS of TSLPv1 and TSLPv2 in vaginal cancer tissues and lymph node metastases of ovarian cancer. (b) Sanger Sequencing of RT-PCR product of TSLPv2.

Figure S3

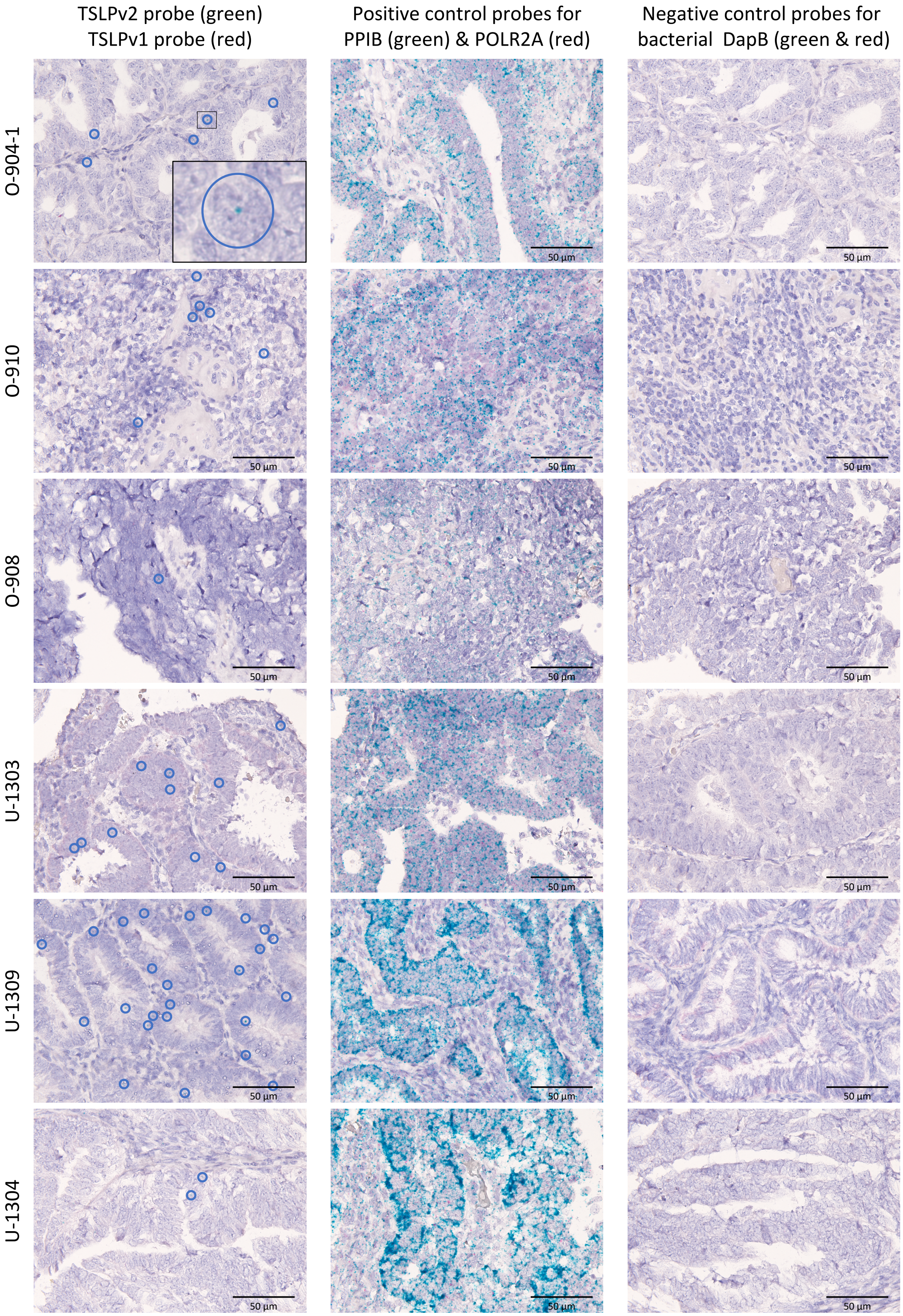

Fig. S4 **mRNA expression of lfTSLP and sfTSLP in selected human EOC and EEC tissues was examined by BaseScope duplex RNA in situ hybridization assay.** Designed BA-Hs-TSLPv1-2zz-st-C2 (red) targeting TSLPv1 and BA-Hs-TSLPv2-3zz-st-C1 (green) targeting TSLPv2 were applied (left column). Blue circles highlighted positive green signals of TSLPv2-specific probe. An inserted picture showed the high magnification of a positive green signal of sfTSLP mRNA expression. Positive control probes (for PIPB in green and POLR2A gene in red; middle column) and negative control probes (for bacterial Dab2 gene in green and red; right column) were also applied. 200x magnification; scale bar 50 µm.

Figure S4

a

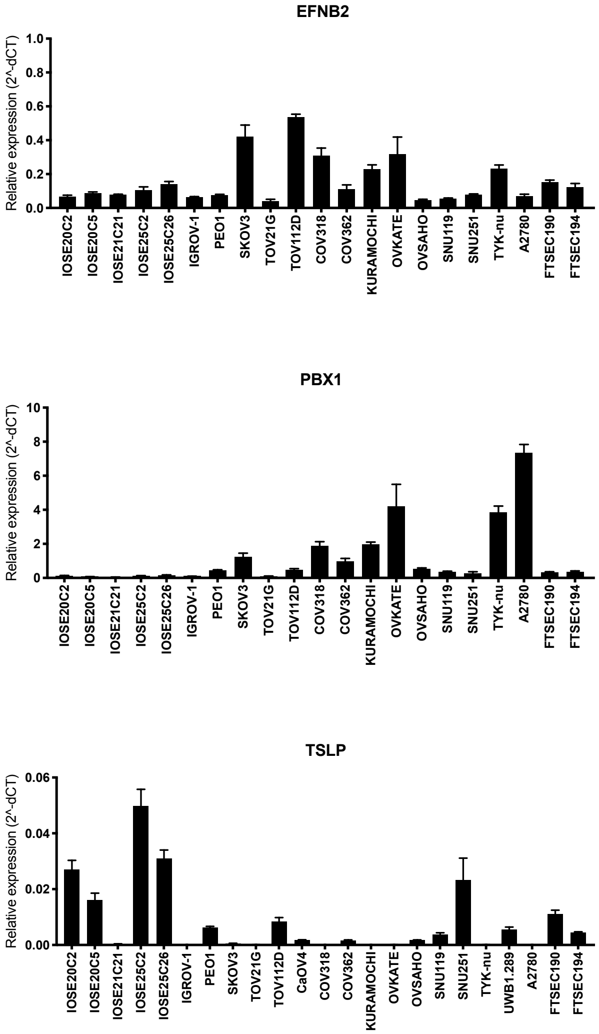

b

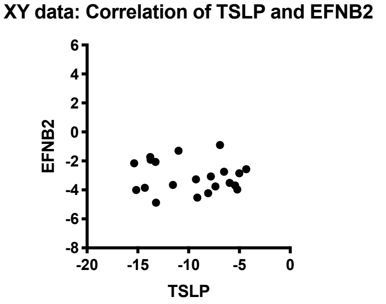

	TSLP vs. EFNB2
Spearman r	
r	-0.03506
95% confidence interval	-0.4705 to 0.4141
P value	
P (two-tailed)	0.88
P value summary	ns
Exact or approximate P value?	Approximate
Significant? (alpha = 0.05)	No
Number of XY Pairs	21

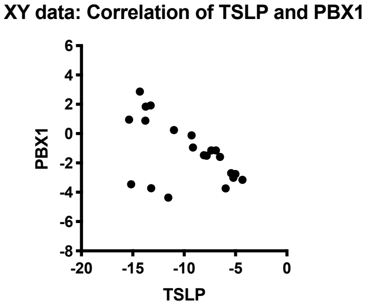

	TSLP vs. PBX1
Spearman r	
r	-0.5013
95% confidence interval	-0.7726 to -0.07527
P value	
P (two-tailed)	0.02
P value summary	*
Exact or approximate P value?	Approximate
Significant? (alpha = 0.05)	Yes
Number of XY Pairs	21

Fig. S5 (a) mRNA expression of EFNB2, PBX1 and TSLP in IOSE, FTSEC and human ovarian cancer cell lines were analyzed by qRT-PCR. (b) Associations between TSLP expression and EFNB1 expression, and between TSLP expression and PBX1 expression were analyzed by Spearman's rank correlation.

Figure S5

a

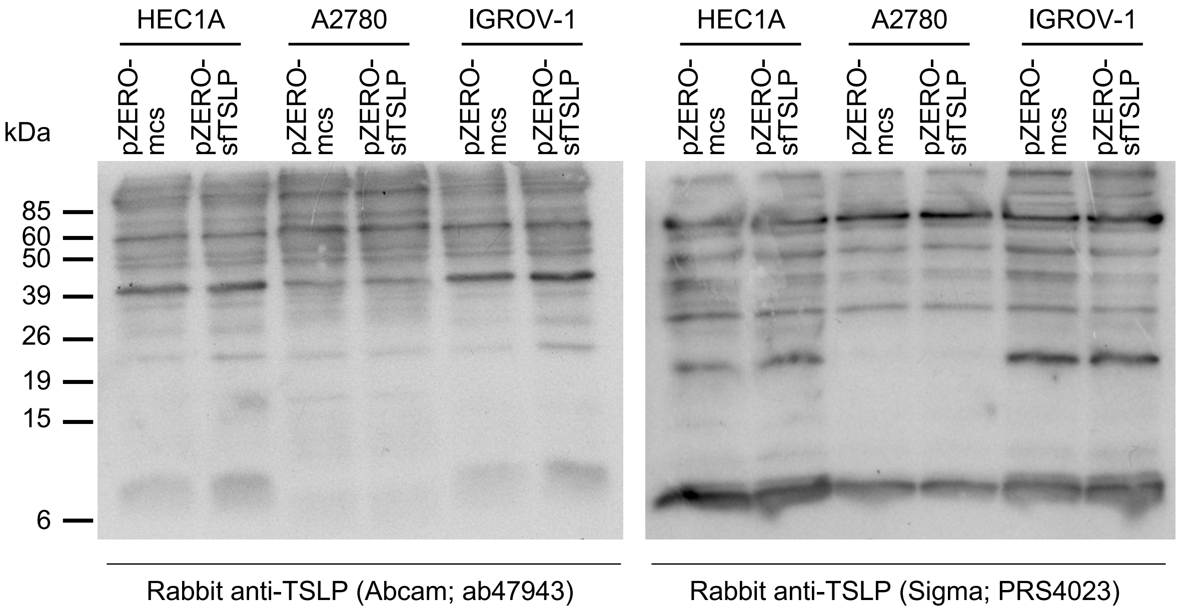

b

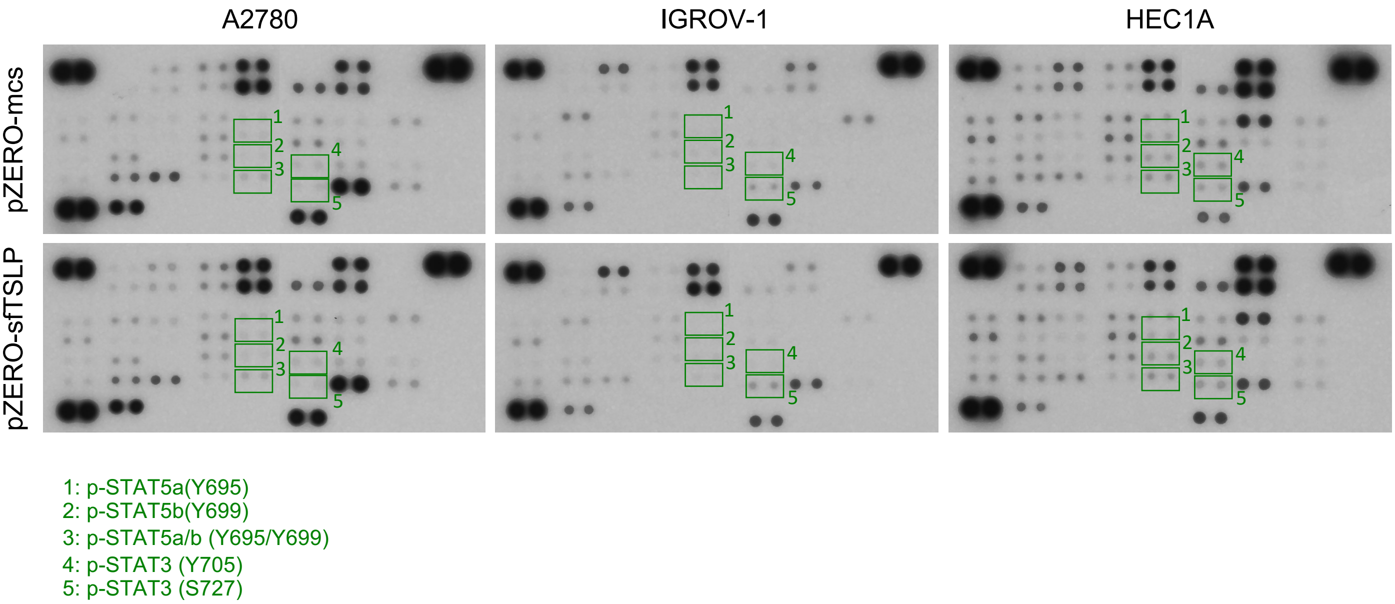

Fig. S6 **Western Blotting of TSLP in ovarian (A2780 and IGROV-1)/endometrial (HEC1A) cancer cells with or without sfTSLP overexpression using two polyclonal antibodies.** (b) **Human Phospho-Kinase Array was performed in human ovarian (A2780 and IGROV-1) and endometrial (HEC1A) cancer cells with or with sfTLSP overexpression**.

Figure S6

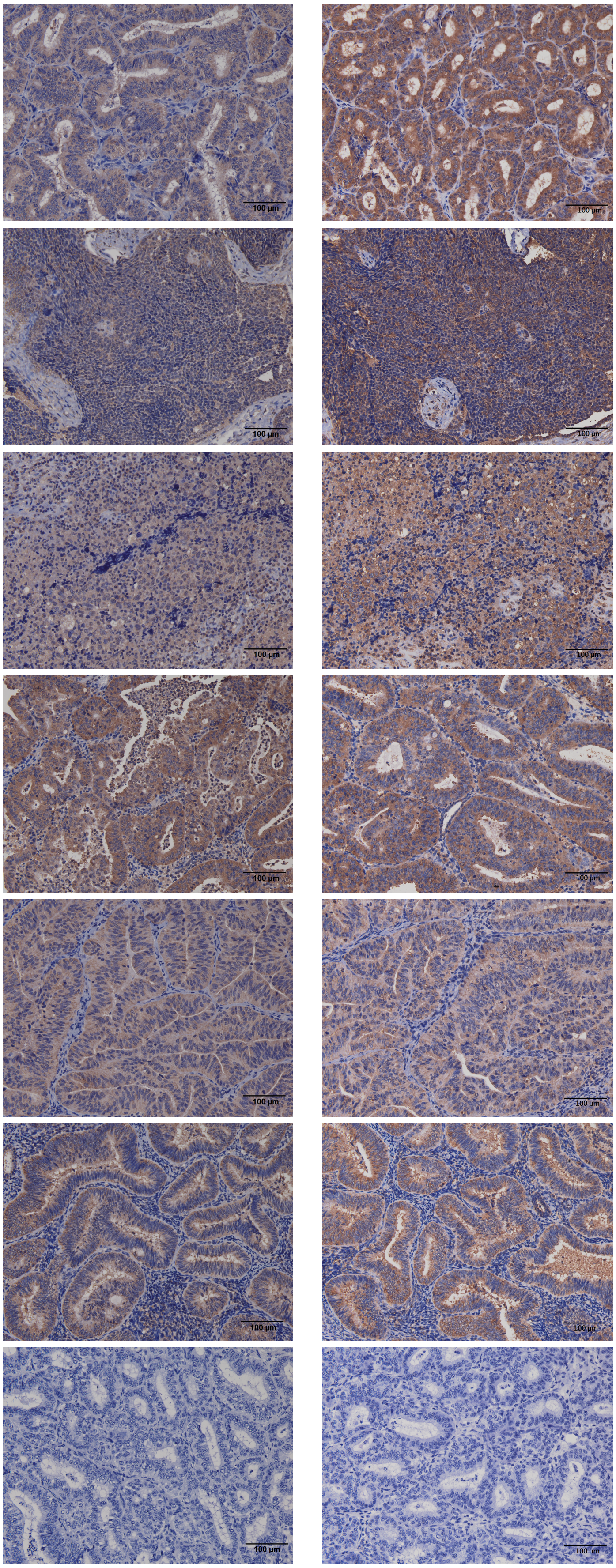

Fig. S7 **Protein expression of TSLP in selected human EOC and EEC tissues was examined by immunohistochemistry using two polyclonal antibodies.**

Figure S7

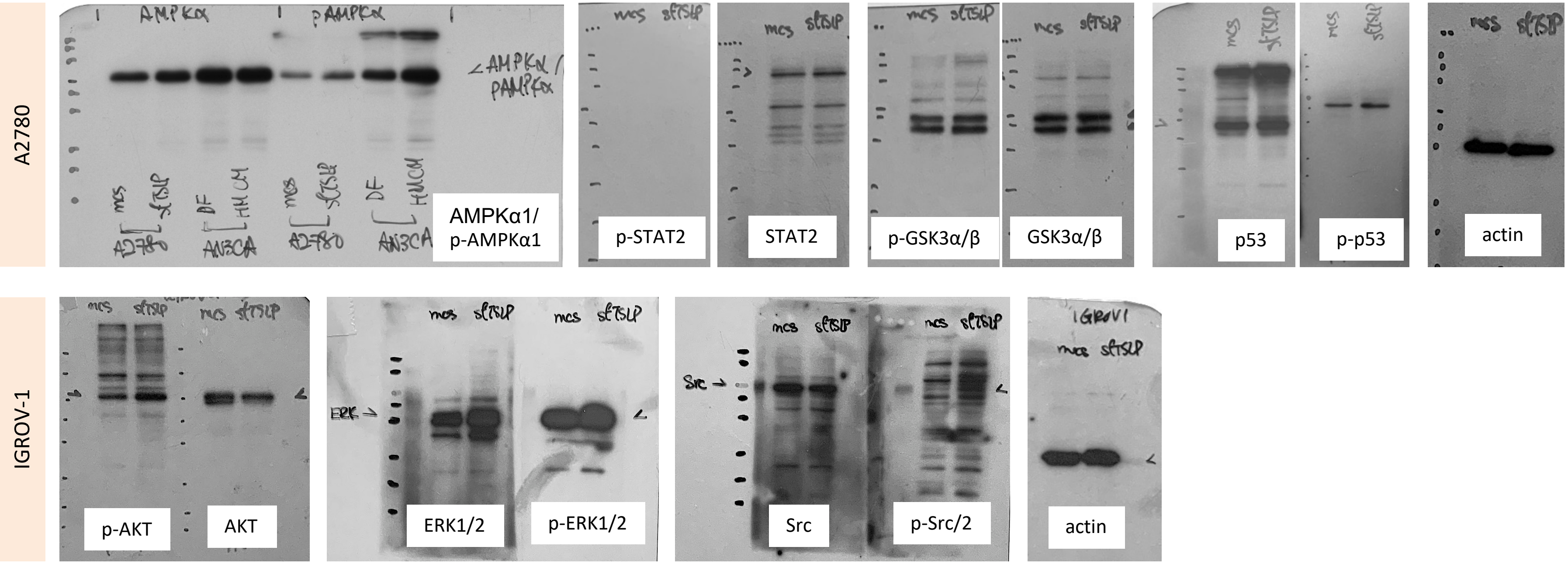

Fig. S8 **The whole Western blots showing all bands and molecular weight markers.**

Figure S8
